# Prognostic value of myocardial perfusion scintigraphy in asymptomatic patients with diabetes mellitus at high cardiovascular risk: 5-year follow-up of the prospective multicenter BARDOT trial

**DOI:** 10.1007/s00259-021-05349-5

**Published:** 2021-04-21

**Authors:** Federico Caobelli, Philip Haaf, Gianluca Haenny, Matthias Pfisterer, Michael J. Zellweger, on behalf of the BARDOT Investigators

**Affiliations:** 1 Clinic of Radiology and Nuclear Medicine, University Hospital Basel and University of Basel, Basel, Switzerland; 2 Cardiovascular Research Institute Basel, Basel, Switzerland; 3 Department of Cardiology, University Hospital Basel and University of Basel, Petersgraben 4, CH-4031 Basel, Switzerland

**Keywords:** Diabetes mellitus, Warranty period, Coronary artery disease, Ergometric stress test, Pharmacologic stress test, Myocardial perfusion SPECT, Cardiovascular risk stratification

## Abstract

**Background:**

The Basel Asymptomatic High-Risk Diabetics’ Outcome Trial (BARDOT) demonstrated that asymptomatic diabetic patients with an abnormal myocardial perfusion scintigraphy (MPS) were at increased risk of major adverse cardiovascular events (MACEs) at 2-year follow-up. It remains unclear whether this finding holds true even for a longer follow-up.

**Methods:**

Four hundred patients with type 2 diabetes, neither history nor symptoms of coronary artery disease (CAD), were evaluated clinically and with MPS. Patients were followed up for 5 years. Major adverse cardiovascular events (MACEs) were defined as all-cause death, myocardial infarction, or late coronary revascularization.

**Results:**

At baseline, an abnormal MPS (SSS ≥ 4 or SDS ≥ 2) was found in 87 of 400 patients (22%). MACE within 5 years occurred in 14 patients with abnormal MPS (16.1%) and in 22 with normal scan (1.7%), *p* = 0.009; 15 deaths were recorded. Patients with completely normal MPS (SSS and SDS = 0) had lower rates of MACEs than patients with abnormal scans (2.5% vs. 7.0%, *p* = 0.032). Patients with abnormal MPS who had undergone revascularization had a lower mortality rate and a better event-free survival from MI and revascularization than patients with abnormal MPS who had either undergone medical therapy only or could not be revascularized (*p* = 0.002).

**Conclusions:**

MPS may have prognostic value in asymptomatic diabetic patients at high cardiovascular risk over a follow-up period of 5 years. Patients with completely normal MPS have a low event rate and may not need retesting within 5 years. Patients with an abnormal MPS have higher event rates and may benefit from a combined medical and revascularization approach.

**Supplementary Information:**

The online version contains supplementary material available at 10.1007/s00259-021-05349-5.

## Introduction

The prospective multicenter Basel Asymptomatic High-Risk Diabetics’ Outcome Trial (BARDOT) showed that asymptomatic patients with diabetes mellitus (DM) at high cardiovascular risk suffer from a higher rate of major adverse cardiovascular events (MACEs) within 2 years in case of abnormal myocardial perfusion scintigraphy (MPS) at baseline. In contrast, a normal MPS allows identifying a subpopulation with a very low likelihood to develop MACEs (2.9%) despite a high cardiovascular risk profile [1]. Although some of the traditional cardiovascular risk factors were predictive of abnormal MPS, even highest risk patients with a normal MPS had a benign prognosis without need for invasive evaluation or therapy [2].

These findings suggest that screening of silent myocardial ischemia in high-risk patients with diabetes mellitus may be useful. To date, screening of all asymptomatic patients with DM remains controversial, as stated in the most recent guidelines [3]. While a meta-analysis including 3299 asymptomatic subjects with DM showed that screening with noninvasive imaging for CAD did not significantly reduce event rates of nonfatal myocardial infarction (MI) and hospitalization for heart failure (HF) [4], the rate of cardiac death and revascularization is higher in patients with abnormal MPS [1].

The relatively low rate of MACEs at short follow-up is conceivably the main reason why the noninvasive assessment of CAD provides controversial benefit on screening asymptomatic patients with diabetes. As such, there is the need for further evidence based on a longer observation. We therefore aimed to investigate the prognostic value of MPS in asymptomatic patients with DM for a 5-year follow-up. The impact of a comprehensive therapeutic approach in these patients was also evaluated.

## Materials and methods

### Patients

Four hundred patients with type 2 diabetes and neither history nor symptoms of CAD were prospectively recruited in the present study as described previously [1]. In short, the high risk of CAD in these patients was documented by end-organ damage (peripheral or carotid occlusive disease, retinopathy, microalbuminuria, and autonomic cardiac neuropathy as measured by Ewing et al. [5]) or by the composite of age older than 55 years, a diabetes duration longer than 5 years, and at least 2 cardiac risk factors (smoking, hypertension, hypercholesterolemia, or positive family history of CAD) in addition to diabetes. Patients older than 75 years, with a life expectancy of less than 3 years, or New York Heart Association (NYHA) functional class IV were excluded. All patients gave written informed consent, and the study protocol was approved by the ethics committee of all 4 participating centers of the Basel Asymptomatic High-Risk Diabetics’ Outcome Trial (BARDOT).

### Study design

**Fig. 1:**
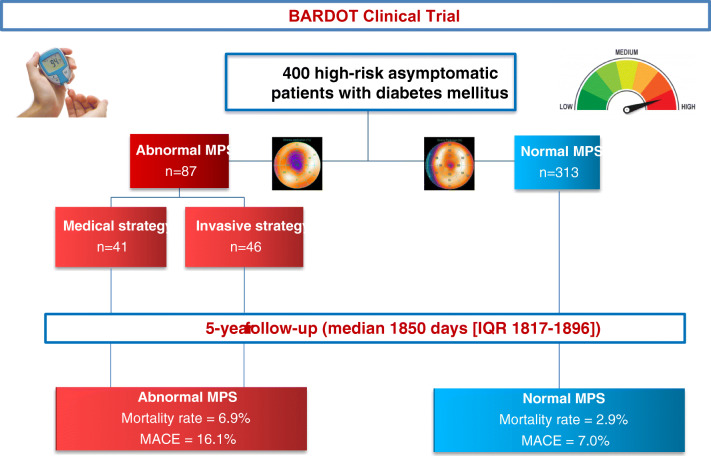
In the BARDOT multicenter trial, 400 asymptomatic diabetic patients with high cardiovascular risk were enrolled and underwent myocardial perfusion SPECT (MPS). Patients with an abnormal MPS were randomized either to an optimal anti-ischemic medical therapy or to the same intense anti-ischemic management including revascularization. The patients were followed up for 5 years. Patients with normal baseline MPS had a lower total mortality rate than patients with an abnormal baseline MPS. Patients with abnormal MPS undergoing an intense anti-ischemic management including revascularization had the most favorable outcome

The design of the present study has been previously described [1] and is shown in Fig. 1. In short, patients underwent clinical visits and rest/stress MPS at baseline and after 2 years and a clinical follow-up after 5 years. If baseline MPS was normal, patients were followed without specific CAD therapy. Patients with abnormal MPS findings were randomly assigned 1:1 to an optimal medical or an optimal medical and whenever possible revascularization strategy.

### Myocardial perfusion scintigraphy

All MPS examinations were performed at the core laboratories of the University Hospital Basel, Switzerland. A single day rest/stress protocol was performed, with the rest examination always performed first, using 400 MBq/800 MBq of ^99m^Tc sestamibi as described earlier [1]. A stress test with a symptom-limited exercise (*n* = 305) or adenosine (*n* = 95) stress and electrocardiographic monitoring was used. Reconstructed and re-oriented images were visually scored by two experienced readers blinded to clinical data using a 17-segment model with a 5-point scale according to American Society of Nuclear Cardiology guidelines (0 = normal perfusion, 1 = mild reduction in counts but not definitively abnormal, 2 = moderate reduction in counts and definitively abnormal, 3 = severe reduction in uptake, and 4 = absent uptake) [6].

Summed scores were calculated globally in the myocardium, summarizing the perfusion scores of the 17 segments. The summed stress score (SSS) equals the sum of the scores for all segments in the stress scan, the summed rest score (SRS) equals the sum of the scores in the rest scan, and the summed difference score (SDS) equals the sum of the differences between SSS and SRS in each segment [7]. We firstly compared patients with unremarkable vs. pathologic MPS, using either SSS of ≥4 or SDS ≥ 2 or both as threshold for CAD [1]. For an exploratory analysis, we further compared patients with a completely normal scan (both SSS and SDS = 0) with the remaining patients with perfusion abnormalities of any degree (SSS > 0 or SDS > 0 or both > 0).

### Endpoints

The endpoint was defined by the occurrence of either all-cause death or of a MACE (i.e., a composite of all-cause death, nonfatal myocardial infarction, and/or revascularization without index revascularization). MI was defined according to current definitions [8] and revascularization as late symptom-driven revascularization (i.e., revascularizations necessary in patients who became symptomatic and remained so despite medical therapy). In patients with multiple or recurrent events (i.e., death, myocardial infarction, revascularization without index revascularization), only the first event was counted as MACE with its respective point in time.

### Statistical analysis

Baseline characteristics of all patients, as well as separately survivors and non-survivors, and patients who were revascularized again during follow-up were compared by unpaired *t* test after normal distribution verification by means of the Kolmogorov-Smirnov test. If the distribution was not normal, the Mann-Whitney *U* test was used. The Fisher exact test was used to compare binary variables.

The rate of death and MACE was compared using Cox proportional hazard models to estimate hazard ratios (HRs) with 95% confidence intervals (CIs) and by Kaplan-Meier curves.

The main analysis for the randomized pilot treatment study part was intention to treat. Differences across groups were assessed by means of Kaplan-Meier analysis. Differences between Kaplan-Meier curves were assessed by using the log-rank test. An “on-treatment” analysis was performed to account for patients in the invasive treatment group who were not revascularized. This is a descriptive, exploratory analysis. Multiple analyses have been performed with the data set, and it is not clear how to calculate the exact family-wise error rate. Consequently, we did not use explicit means to control the family-wise error rate. Accordingly, we did not treat *p* values as having a threshold indicating “significance,” but rather as a tool, supplementary to the effect estimates (hazard ratios) and their confidence intervals. Conclusions are drawn not for each “statistical test” based on *p* < 0.05 but on the cumulating evidence and effect sizes from all the models. Analysis was performed with SPSS for Microsoft Windows v. 22 (IBM, USA).

## Results

### Characteristics of patients

Baseline characteristics of the 400 patients are shown in Table 1. Enrolled patients were predominantly male (69%) and had a median diabetes duration of 9 years, and 85% presented with more than one diabetic end-organ damage. Patients who died over the follow-up period of 5 years more often presented with shortness of breath, higher resting heart rates, ECG changes during ergometry, and perfusion abnormalities on MPS. There was a tendency to higher occurrence of autonomic neuropathy in patients who died. There were no differences between survivors and deceased patients regarding age, gender, diabetes duration/HbA1c end-organ damage, medication, smoking, or lipid status (Table 1). Higher SSS and SRS were found in patients who died during follow-up, while SDS were not different. Conversely, higher SDS (but not SSS and SRS) were reported in patients undergoing revascularization during the 5-year follow-up.

**Table 1:** Baseline characteristics of the 400 patients

	All patients (*n* = 400)	Patients who died (*n* = 15)	Survivors (*n* = 385)	*p* value	Revascularization within 5 years (except for index) (*n* = 23)	No revascularization within 5 years (except for index) (*n* = 377)	*p* value
Age, years	63 (58–68)	67 (58–73)	63 (58–68)	0.093	65 (58–68)	63 (58–68)	0.672
Male gender (%)	68.8	80.0	68.3	0.388	82.6	67.9	0.140
Diabetes duration, years	9 (5–15)	10 (6–20)	9 (5–15)	0.335	10 (8–16)	9 (5–15)	0.110
BMI, kg/m^2^	29.7 (26.6–33.5)	28.1 (25.8–31.7)	29.8 (26.6–33.6)	0.463	27.2 (25.80–30.0)	30.0 (26.6–33.7)	0.024
End-organ damage (%)							
Retinopathy	23.3	6.7	23.9	0.121	13.0	23.9	0.233
Polyneuropathy	48.5	57.1	48.3	0.516	43.5	48.8	0.611
Nephropathy	46.3	57.1	46.0	0.410	60.9	45.4	0.151
Autonomic neuropathy	45.0	71.4	45.1	0.052	31.8	45.5	0.209
Peripheral artery disease	14.5	26.7	14.1	0.174	17.4	14.4	0.689
Stroke/TIA	8.8	6.7	8.8	0.771	13.0	8.5	0.453
Patients with ≥1 of listed end-organ damages	85.3	86.7	85.2	0.875	87.0	87.3	0.965
Smoking (%)	20.8	26.7	20.5	0.565	21.7	20.7	0.904
Shortness of breath (%)	46.8	73.3	45.7	0.035	30.4	47.7	0.106
Resting heart rate, beats/min	74 (66–82)	84 (67–97)	74 (66–81)	0.048	76 (66–88)	74 (66–81)	0.734
Systolic blood pressure, mm Hg	138 (124–150)	130 (120–148)	138 (124–150)	0.565	140 (130–150)	137 (122–150)	0.375
HbA1c	7.1 (6.5–7.8)	5.7 (5.4–7.4)	7.1 (6.5–7.8)	0.718	7.0 (6.7–8.0)	7.1 (6.5–7.8)	0.013
Microalbuminuria (%)	45.8	57.1	45.5	0.389	60.9	44.9	0.137
Estimated glomerular filtration rate (ml/min/1.73 m^2^)	110 (84–136)	93 (71–130]	111 (84–137)	0.148	103 (88–124)	111 (84–137)	0.333
Total cholesterol, mmol/l	4.6 (3.9–5.3)	4.2 (3.6–5.2)	4.6 (3.9–5.3)	0.295	4.8 (3.8–6.0)	4.6 (3.9–5.3)	0.739
LDL cholesterol, mmol/l	2.4 (1.8–3.0)	1.9 (1.7–3.0)	2.4 (1.9–3.0)	0.280	2.8 (2.0–3.3)	2.4 (1.8–3.0)	0.596
BNP, ng/l, median	37 (19–78)	39 (27–203)	37 (18–76)	0.311	33 (15–58)	37 (19–79)	0.386
Medication (%)							
Antiplatelet drugs	52.5	66.7	51.9	0.263	73.9	51.2	0.034
Oral anticoagulants	4.8	13.3	4.4	0.111	0.0	5.0	0.270
Beta blocker	32.0	40.0	31.7	0.498	34.8	31.8	0.768
Calcium antagonist	25.3	20.0	25.5	0.633	17.4	25.7	0.372
Nitrates	0.5	0.0	0.5	0.780	0.0	0.5	0.726
Statins	57.3	66.7	56.9	0.452	47.8	57.8	0.347
ACE-I/ARB	76.5	93.3	75.8	0.117	78.3	76.4	0.837
Diuretics	48.8	60.0	48.3	0.374	56.5	48.3	0.442
Oral glucose-lowering therapy	80.0	80.0	80.0	1.000	82.6	79.8	0.747
Insulin	50.2	66.7	49.6	0.195	47.8	50.4	0.811
ECG q-waves (%)	4.8	13.3	4.4	0.111	0	5.0	0.270
Symptoms during stress test (%)	13.0	20.0	12.7	0.411	8.7	13.3	0.527
ECG changes during stress test (%)	10.0	26.7	9.4	0.028	21.7	9.30	0.053
SRS/% myocardium, median	0 (0–1)	0 (0–5)	0 (0–1)	0.032	0 (0–1)	0 (0–1)	0.912
SSS/% myocardium, median	0 (0–2)	2 (0–8)	0 (0–1)	0.026	0 (0–7)	0 (0–1)	0.142
SDS/% myocardium ischemic, median	0 (0–0)	0 (0–0)	0 (0–0)	0.618	0 (0–6)	0 (0–0)	0.021
Transient ischemic dilatation (%)	4.5	6.7	4.4	0.680	4.3	4.5	0.971
Left ventricular ejection fraction at rest, %	58 (52–64)	55 (47–63)	58 (52–64)	0.143	57 (53–62)	58 (52–64)	0.488
Left ventricular ejection fraction post-stress, %	58 (52–65)	55 (37–62)	58 (52–65)	0.052	57 (52–64)	58 (52–66)	0.441

Values are median (inter-quartile range (IQR)). All variables apart from total cholesterol were non-normally distributed. *ACE-I/ARB* angiotensin-converting enzyme inhibitor/angiotensin receptor blocker, *BMI* body mass index, *BNP* brain natriuretic peptide, *ECG* electrocardiogram, *HDL* high-density lipoprotein, *HbA1c* hemoglobin A1c, *MPS* myocardial perfusion single-photon emission computed tomography, *NYHA* New York Heart Association, *SDS* summed difference score, *SSS* summed stress score, *TIA* transient ischemic attack

*p* values were calculated with the use of a Mann-Whitney *U* test for quantitative variables and a chi-square test for qualitative variables. Resting heart rate, shortness of breath, and ECG changes during stress were predictors of all-cause death. Also SSS and SRS were significant predictors, while SDS failed to show significance. Of note, patients undergoing revascularization (thus with a better outcome) had higher SDS values

### Death, myocardial infarction, and revascularization during 5-year follow-up

During the 5-year follow-up period (median 1840 days [IQR 1817–1896]), 15 deaths (3.8%) were recorded and 11 patients (2.8%) suffered from MI (1 STEMI, 10 NSTEMI). Twelve patients experienced more than one event during the 5-year follow-up. At the outset of the BARDOT study (1), 87 patients had an abnormal baseline MPS and were randomized to a medical strategy (*n* = 41) or invasive strategy arm (*n* = 46, intention to treat). Thirty patients (65%) of the invasive strategy arm of the study had been successfully revascularized at baseline, while none of the medical strategy arm underwent an early revascularization. Overall median time between MPS and index revascularization amounted to 42 days [IQR 27–54]; there was no significant difference between index percutaneous coronary intervention and index bypass surgery (*p* value for comparison = 0.081).

In addition to these index revascularizations, 23 more coronary revascularizations have been performed during the 5-year follow-up period. Fourteen of these revascularizations were done in patients with normal baseline MPS (14/313, 4.5%) and 9 with abnormal baseline MPS (9/87, 10.3%; *p* value 0.037). Of these 9 patients, 7 were in the medical strategy arm. Two of these 9 patients were in the invasive strategy arm and were thus twice revascularized (index revascularization and true follow-up revascularization). For a juxtaposition of (1) all patients, (2) patients who survived (*n* = 385), and (3) patients who died (all-cause mortality, *n* = 15) regarding the occurrence of death, myocardial infarction, revascularization, and cardiac hospitalizations during the 5-year follow-up period, see also Supplement, Table 1.

### Prognostic value of MPS for the prediction of all-cause death during 5-year follow-up

**Fig. 2:**
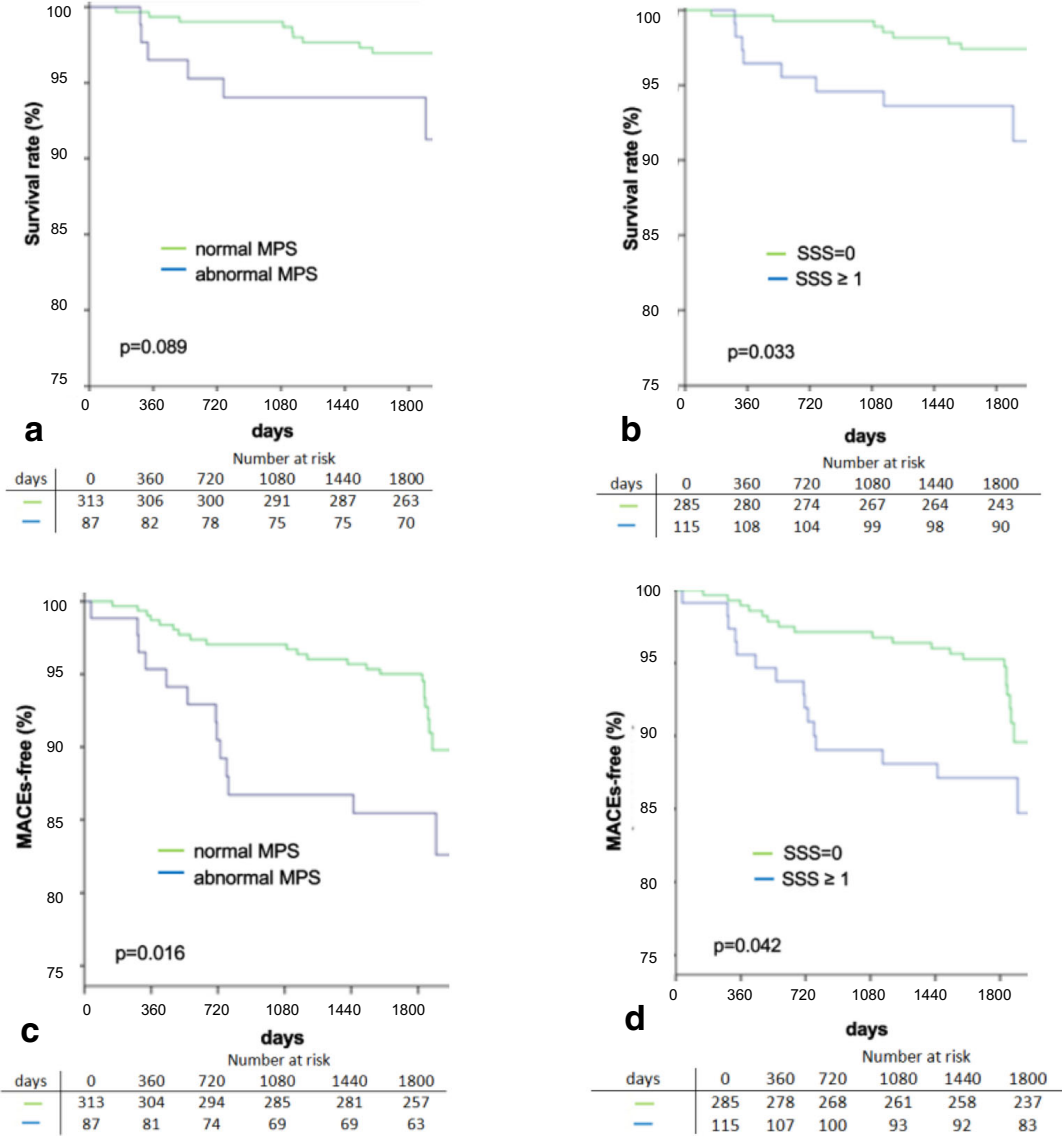
**a**–**d** Kaplan-Meier curve analysis for survival in (**a**) normal (SSS < 4 and SDS < 2) vs. abnormal MPS (SSS ≥ 4 or SDS ≥ 2); (**b**) (SRS/SSS = 0) vs. (SRS/SSS ≥ 1); survival free from myocardial infarction or revascularization (without index) in (**c**) normal vs. abnormal MPS and (**d**) (SRS/SSS = 0) vs. (SRS/SSS ≥ 1). MPS, myocardial perfusion scintigraphy; MI, myocardial infarction; SRS, summed rest score; SSS, summed stress score; SDS, summed difference score

**Fig. 3:**
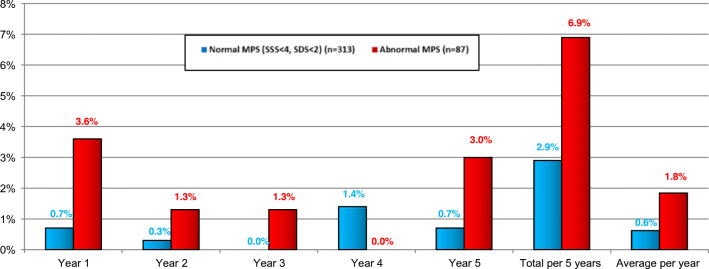
Mortality rate per year, total mortality rate per 5 years, and average mortality per year stratified for normal and abnormal MPS. MPS, myocardial perfusion scintigraphy; SSS, summed stress score; SDS, summed difference score. The percentages reported are death rates in the respective years for both treatment groups. Death rates are based on number at risk as presented in Fig. 2. The aim is not to estimate the event rates per se but to compare among the treatment groups. Total per 5 years refers to the cumulative event rate during the 5-year follow-up

Eighty-seven patients (22%) showed perfusion defects of various degree (SSS ≥ 4 and/or SDS ≥ 2), while 313 (78%) had a normal baseline perfusion scintigraphy scan (SSS < 4, SDS < 2); 285 (71%) patients had a completely normal scan (SSS = 0 and SDS = 0), which made up the majority (285/313, i.e., 91%) of all normal scans. There was an overall tendency to better survival in patients with normal and a better survival in patients with completely normal baseline MPS (Fig. 2a, b, Table 2). This results in a total 5-year death rate of 2.9% for patients with normal MPS vs. 6.9% with abnormal MPS (*p* = 0.081), respectively, and 2.5% of patients with completely normal scans vs. 7.0% with perfusion abnormalities of any degree (i.e., with either SSS > 0 or SDS > 0 or both, *p* = 0.032, Fig. 2). The average death rate per year was 0.6% in both normal and completely normal MPS patients, while patients with abnormal scan had an average death rate of 1.8% per year (Fig. 3). Cox proportional hazard analysis (Supplement, Table 2) displayed that baseline SRS and SSS were predictors of all-cause death (*p* = 0.048) and a completely normal scan is consistent with a lower risk of death (*p* = 0.040) (Supplement, Table 2, Fig. 2b). The prognostic accuracy of an abnormal MPS (SSS ≥ 4 and SDS ≥ 2) to predict all-cause death did not reach statistical significance (*p* = 0.090) (Supplement, Table 2, Fig. 2).

**Table 2:** Myocardial perfusion scintigraphy for the prediction of death, myocardial infarction, and revascularization during the 5-year follow-up

	Normal MPS (SSS < 4, SDS < 2)	Abnormal MPS (SSS ≥ 4 and/or SDS ≥ 2)	Sum	*p* value	Completely normal MPS (SSS = 0 and SDS = 0)	MPS with SSS ≥ 1 or SDS ≥ 1	Sum	*p* value
Death	9 (2.9%)	6 (6.9%)	15	0.081	7 (2.5%)	8 (7.0%)	15	0.032
Survivors	304 (97.1%)	81 (93.1%)	385		278 (97.5%)	107 (93.0%)	385	
Death or myocardial infarction (MI)	17 (5.4%)	7 (8.0%)	24	0.364	15 (5.3%)	9 (7.8%)	24	0.329
Survivors without MI	296 (94.6%)	80 (92.0%)	376		270 (94.7%)	106 (92.2%)	376	
Death, MI, or revascularization (without index revascularization)	22 (7.0%)	14 (16.1%)	36	0.009	20 (7.0%)	16 (13.9%)	36	0.029
Survivors without MI or revascularization (without index revascularization)	291 (93.0%)	73 (83.9%)	364		265 (93.0%)	99 (86.1%)	364	

*MPS* myocardial perfusion scintigraphy, *SSS* summed stress score, *SDS* summed difference score, *MI* myocardial infarction

### Prognostic value of MPS for the prediction of all-cause death, myocardial infarction, or revascularization (without index revascularization) during 5-year follow-up

Thirty-six patients did not survive free from myocardial infarction or revascularization (without index revascularization) during 5-year follow-up. A normal MPS during follow-up was associated with a lower rate of MACEs compared to patients with evidence of perfusion abnormalities (22/313, 7.0% vs. 14/87, 16.1%, *p* = 0.009). The same held true for patients with a completely normal scan (20/285, 7.0% vs. 16/115, 13.9%, *p* = 0.029, Fig. 2c, d, Table 2). Consistently, Cox proportional hazard analysis (Supplement, Table 2) showed that patients with an abnormal MPS (SSS ≥ 4 and/or SDS ≥ 2) had a higher risk of all-cause death, myocardial infarction, or revascularization (*p* = 0.011), while a completely normal scan was associated with a very low probability of MACEs (*p* = 0.032).

### Impact of therapeutic strategy on prognosis

Thirty patients (65%) of the invasive strategy arm of the study (*n* = 46) had been successfully revascularized (21 by percutaneous coronary intervention, 9 by bypass surgery); 16 patients (35%) of the invasive strategy arm either refused the angiography or revascularization had not been feasible because of unfavorable anatomy [1].

**Fig. 4:**
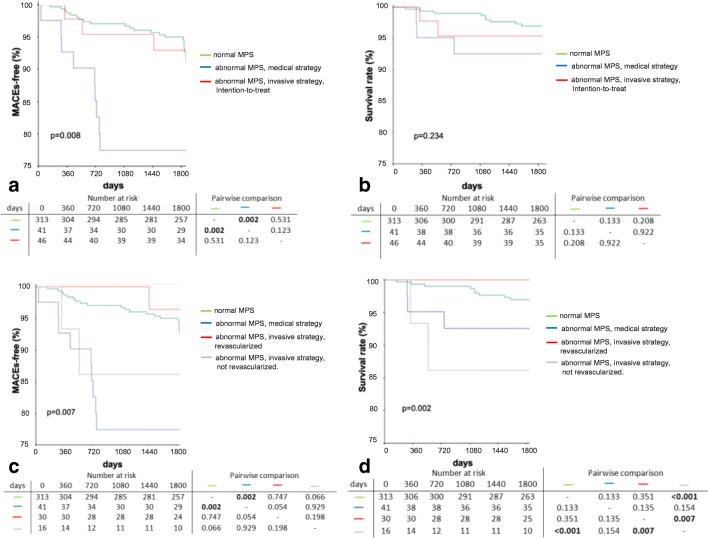
**a**–**d** Kaplan-Meier curve analysis for survival in (**a**) normal (SSS < 4 and SDS < 2) vs. abnormal MPS (SSS ≥ 4 or SDS ≥ 2) with medical vs. invasive strategy (intention to treat), (**b**) normal vs. abnormal MPS with medical vs. invasive strategy (and revascularized) vs. invasive strategy (but not revascularized); survival free from myocardial infarction or revascularization (without index) in (**c**) normal vs. abnormal MPS with medical vs. invasive strategy (intention to treat) and (**d**) normal vs. abnormal MPS with medical vs. invasive strategy (and revascularized) vs. invasive strategy (but not revascularized). MPS, myocardial perfusion scintigraphy; MI, myocardial infarction

In an intention-to-treat Kaplan-Meier curve analysis, patients with abnormal MPS randomized to medical strategy had worse outcome with respect to death, MI, or revascularization (without index revascularization) compared to both patients with normal MPS and patients with abnormal MPS and subsequent invasive strategy (*p* = 0.008) (Fig. 4a). For overall survival, the difference between the three groups did not reach statistical significance (*p* = 0.234) (Fig. 4b).

In an as-treated Kaplan-Meier curve analysis, patients with abnormal MPS who had undergone revascularization had a lower mortality rate and a better event-free survival from MI and revascularization than patients who had undergone medical therapy only and patients who either refused invasive evaluation or in patients in whom revascularization was not feasible (*p* = 0.007, Fig. 4c). Of note, no deaths were recorded among those patients with abnormal MPS in the invasive strategy arm and successful index revascularization (Fig. 4d).

## Discussion

The BARDOT trial showed that MPS allows for robust risk stratification in asymptomatic diabetic patients at high cardiovascular risk up to 2 years after testing [1]. Patients with a normal MPS at baseline had similar mortality rates as the normal population. In contrast, patients with an abnormal MPS had significantly higher event rates. In patients with abnormal MPS, patients who underwent protocol revascularization in addition to optimal medical therapy had lower event rates than patients with medical therapy only.

The recently published guidelines of the European Society of Cardiology (ESC) do not recommend routine screening of asymptomatic diabetic patients, since the impact of routine screening of CAD in asymptomatic DM and no history of CAD has shown no differences in cardiac outcomes [3].

Our work expands the current knowledge, providing insights into the long-term predictive role of MPS in the assessment of asymptomatic high-risk diabetic patients. Three major findings can be summarized, when patients are followed up for at least 5 years.

First, it was confirmed that a reduced rate of MACEs in the selected populations also pertains to a longer follow-up if baseline MPS is unremarkable. The clinical implications of these findings are relevant, as the role of MPS as a screening test to identify those patients at increased risk of developing MACEs on a relatively long follow-up is controversial. The DYNAMIT trial [9] showed no significant difference between the screening and the usual care group for the primary outcome, defined as all-cause death, myocardial infarction, or heart failure. The DIAD trial showed no difference in the occurrence of cardiac death or nonfatal myocardial infarction between patients screened and not screened with MPS [10]. The controversy may be related to the difference in the study design and to the different patients’ population. In fact, patients in the DIAD study had a lower cardiovascular risk compared to our population: besides having a lower rate of end-organ damage, only 6% of patients in the DIAD trial had perfusion defects of at least 5% of the myocardium which was required in all BARDOT patients. Moreover, the randomization in the DIAD study was performed to select those patients willing to undergo an MPS, while our randomization followed the results of MPS, thus comprising all patients with perfusion abnormalities. Then, the therapy decision in case of perfusion abnormalities in the DIAD trial was left at the discretion of the treating physician, while the decision algorithm in our study was established per protocol [1]. Finally, outcome measures of DIAD were restricted to cardiac death or MI, while we also included revascularization as cardiac event, which also needs to be considered if CAD progression is assessed.

As such, we confirm the complementarity of our and previous published studies: a general screening of all asymptomatic patients with diabetes may not be needed but is of prognostic value in asymptomatic patients with diabetes at high coronary risk, as also underlined in the most recent guidelines [3]. Of note, scintigraphic data showed a prognostic significance in our patients’ population, while other important clinical variables did not. Specifically, shortness of breath was one of the few clinical variables associated with a worse survival rate, while, e.g., autonomic impairment and higher heart rate showed, if at all, only a weak tendency toward a worse outcome. These results are consistent with a previous report on 1737 patients with diabetes mellitus, wherein the rate of death and/or myocardial infarction was three times worse in diabetic patients with shortness of breath and MPS findings were strongly predictive of outcome [11].

The second point is the warranty period of a completely normal MPS in asymptomatic diabetic patients at high cardiovascular risk. When to (re)-evaluate cardiac risk is an important question. When should a diabetic patient with a normal scan be retested? In this context, a first retrospective study postulated a warranty period of 1 to 3 years in patients with a normal MPS [12]. Acampa et al. [13] also expanded on this topic in a population of both symptomatic and asymptomatic diabetic patients, showing that even in case of a normal MPS diabetic patients are at higher risk for cardiac events and that the warranty period of a normal stress MPS varies according to diabetic status and post-stress LVEF. The same group [14] also showed that in asymptomatic diabetics, post-stress LVEF ≤45% and a large stress-induced ischemia are predictors of a worse risk over time at long-term follow-up. Our study provides more evidence on this important topic. Independently from diabetic status and clinical manifestations, MPS proved effective in predicting a more favorable outcome, and patients with a completely normal MPS had an excellent prognosis over 5 years with mortality rates (0.6%/year) similar to that of the normal population. As such, it may be maintained that there is no need for retesting for CAD within 5 years even in patients with diabetes at high risk in case of a completely normal scan.

Third, we confirm the better outcome of patients treated with a combined medical and invasive approach also for a longer follow-up. The question as to whether an invasive approach should be pursued in patients with detectable ischemia is a matter of debate. While some reports showed a benefit of the invasive strategy over medical therapy alone [15, 16], the recent ISCHEMIA Trial [17] reported no evidence of benefit of an initial invasive strategy over a conservative one with regard to the occurrence of MACEs or death from any cause in patients with stable angina. It should be noted that none of these papers focused on diabetic patients at high cardiovascular risk. Conversely, the BARI-2D trial, focused on diabetic patients, showed reduced rate of MACEs in patients revascularized with CABG (22.4%) compared to those treated with medical therapy approach (30.5%, *p* = 0.01), thus suggesting a benefit from the revascularization strategy in diabetic patients [18]. Furthermore, a retrospective study featuring asymptomatic diabetic patients showed an improved survival rate in patients undergoing revascularization compared to those treated medically [19].

The results of our study support the concept that an invasive/revascularization strategy may be preferred in asymptomatic diabetic patients with scintigraphic evidence of CAD in view of their better outcome. In fact, patients undergoing revascularization in our population had a favorable outcome, which was equivalent to those patients with an unremarkable MPS, thus bearing importance in clinical practice for the choice of the most appropriate therapeutic option.

### Limitations

First, the study was planned before calcium scoring and CT-based coronary angiography (CCTA) were routinely used, as such the impact of coronary calcification and/or significant stenosis as detected on CCTA cannot be assessed. Furthermore, MPS protocol did not use the most recent technological improvements on image reconstruction such as attenuation and scatter correction. However, prone images were employed to increase the specificity of the technique, and the observers’ experience (>10 years) makes it unlikely that the use of the most modern protocols would significantly have modified our results.

## Conclusion

MPS has prognostic value in asymptomatic diabetic patients at high cardiovascular risk over a follow-up period of 5 years. Patients with a completely normal MPS have a low event rate and do not need retesting within 5 years. Patients with an abnormal MPS have higher event rates and may benefit from a combined medical and revascularization approach.

## Supplementary information

ESM 1 (DOCX 23 kb)
