# Supplementary material for: Prognostic value of myocardial perfusion scintigraphy in asymptomatic patients with diabetes mellitus at high cardiovascular risk: 5-year follow-up of the prospective multicenter BARDOT trial
Source: Eur J Nucl Med Mol Imaging. 2021 Apr 21;48(11):3512–21. doi: 10.1007/s00259-021-05349-5 (PMC8440314; doi:10.1007/s00259-021-05349-5)
Supplement: Supplementary file 1 — (DOCX 23 kb) [file 259_2021_5349_MOESM1_ESM.docx]

**Supplement Data**

| **Supplement, Table 1** | **Occurrence of death, myocardial infarction, revascularization and cardiac hospitalizations during the 5-year follow-up period** | | | |
| --- | --- | --- | --- | --- |
|  | **All patients**  **(n=400)** | **Survivors**  **(n=385)** | **Patients who died**  **(n=15)** | **p value** |
| Death | 15 (3.8%) | 0 (0%) | 15 (100%) | - |
| Cardiac death | 5 (1.3%) | 0 (0%) | 5 (33.3%) | - |
| Myocardial infarction | 11 (2.8%) | 9 (2.3%) | 2 (13.3%) | 0.011 |
| STEMI | 1 (0.3%) | 1 (0.3%) | 0 (0%) | 0.843 |
| NSTEMI | 10 (2.5%) | 8 (2.1%) | 2 (13.3%) | 0.006 |
| Death or myocardial infarction | 24 (6.0%) | 9 (2.3%) | 15 (100%) | (<0.001) |
| **Revascularization (index)** | 30 (7.5%) | 30 (7.8%) | 0 (0%) | 0.261 |
| Interventional revasculariziation | 21 (5.3%) | 21 (5.5%) | 0 (0%) | 0.353 |
| Surgical revascularization | 9 (2.3%) | 9 (2.3%) | 0 (0%) | 0.549 |
| **Revascularization (without index)** | 23 (5.8%) | 21 (5.5%) | 2 (13.3%) | 0.198 |
| Interventional revasculariziation | 17 (4.3%) | 16 (4.2%) | 1 (6.7%) | 0.636 |
| Surgical revascularization | 6 (1.5%) | 5 (1.3%) | 1 (6.7%) | 0.093 |
| Cardiac hospitalization | 46 (11.5%) | 42 (10.9%) | 4 (26.7%) | 0.061 |

(N)STEMI = (non) ST elevation myocardial infarction; two patients with index revascularizations were revascularized again during the follow-up period and also died: one person died 573 days after interventional revascularization and one person died 43 days after surgical revascularization.

| **Supplement, Table 2** | **Univariate Cox proportional hazard analysis** | | | |
| --- | --- | --- | --- | --- |
|  | **Prediction of all-cause death during 5-year follow-up** | | **Prediction of all-cause death, myocardial infarction and revascularization (without index) during 5-year follow-up** | |
| **Variable** | **Hazard ratio**  **(95% CI)** | **p-value** | **Hazard ratio**  **(95% CI)** | **p-value** |
| Age, years | 1.072 (0.989-1.161) | 0.091 | 1.041 (0.990-1.093) | 0.114 |
| Shortness of breath | 3.266 (1.022-10.436) | 0.046 | 1.021 (0.514-2.027) | 0.953 |
| Resting heart rate | 1.061 (1.017-1.108) | 0.007 | 1.027 (0.998-1.057) | 0.072 |
| ECG changes during stress | 3.525 (1.067-11.642) | 0.039 | 2.964 (1.248-7.043) | 0.014 |
| Physical stress during MPS | 0.851 (0.265-2.738) | 0.787 | 1.099 (0.483-2.500) | 0.821 |
| *Myocardial perfusion scintigraphy (baseline)* |  |  |  |  |
| Abnormal MPS (SSS≥4 and/or SDS≥2) | 2.502 (0.865-7.234) | 0.090 | 2.537 (1.238-5.199) | 0.011 |
| Completely normal scan (SSS=0 and SDS=0 (baseline)) | 0.337 (0.119-0.951) | 0.040 | 0.467 (0.233-0.937) | 0.032 |
| SSS (baseline) | 1.089 (1.001-1.185) | 0.048 | 1.218 (1.071-1.385) | 0.003 |
| SDS (baseline) | 1.060 (0.868-1.295) | 0.567 | 1.279 (0.282-5.801) | 0.749 |
| Transient ischemic dilatation | 1.546 (0.192-12.454) | 0.682 | 0.992 (0.943-1.043) | 0.747 |
| LVEF drop (post stress EF – rest EF) | 0.995 (0.940-1.053) | 0.865 | 1.041 (0.990-1.093) | 0.114 |

SRS = summed rest score; SSS = summed stress score; SDS = summed difference score; SRS, SSS and SDS are used as continuous variables; (LV)EF = (left ventricular) ejection fraction. Due to the relatively low number of events, we refrained from performing a multivariate cox proportional analysis for all-cause death. Regarding MACE: the univariately significant variable “ECG changes during stress” did not remain significant neither when analyzed together with “Abnormal MPS”, “Completely normal scan” nor with “SSS (baseline)”. Due to the low number of events, identifying deviations from the proportional hazards was difficult. However, no clear indication of violation was noted (ie. no crossing hazard functions).
